# Spatially structured environmental filtering of collembolan traits in late successional salt marsh vegetation

**DOI:** 10.1007/s00442-015-3345-z

**Published:** 2015-05-23

**Authors:** Lina A. Widenfalk, Jan Bengtsson, Åsa Berggren, Krista Zwiggelaar, Evelien Spijkman, Florrie Huyer-Brugman, Matty P. Berg

**Affiliations:** Department of Ecology, Swedish University of Agricultural Sciences, P.O. Box 7044, SE-75007 Uppsala, Sweden; Department of Ecological Sciences, VU University, Amsterdam, De Boelelaan 1085, 1081 HV Amsterdam, The Netherlands; Community and Conservation Ecology Group, Center for Ecological and Evolutionary Studies, University of Groningen, Nijenborgh 7, 9747 AG Groningen, The Netherlands

**Keywords:** Diversity, Functional trait, Scale, Spatial configuration, Springtail, Variance partitioning

## Abstract

Both the environment and the spatial configuration of habitat patches are important factors that shape community composition and affect species diversity patterns. Species have traits that allow them to respond to their environment. Our current knowledge on environment to species traits relationships is limited in spite of its potential importance for understanding community assembly and ecosystem function. The aim of our study was to examine the relative roles of environmental and spatial variables for the small-scale variation in Collembola (springtail) communities in a Dutch salt marsh. We used a trait-based approach in combination with spatial statistics and variance partitioning, between environmental and spatial variables, to examine the important ecological factors that drive community composition. Turnover of trait diversity across space was lower than for species diversity. Most of the variation in community composition was explained by small-scale spatial variation in topography, on a scale of 4–6 m, most likely because it determines the effect of inundation, which restricts where habitat generalists can persist. There were only small pure spatial effects on species and trait diversity, indicating that biotic interactions or dispersal limitation probably were less important for structuring the community at this scale. Our results suggest that for springtails, life form (i.e. whether they live in the soil or litter or on the surface/in vegetation) is an important and useful trait to understand community assembly. Hence, using traits in addition to species identity when analysing environment–organism relationships results in a better understanding of the factors affecting community composition.

**Electronic supplementary material:**

The online version of this article (doi:10.1007/s00442-015-3345-z) contains supplementary material, which is available to authorized users.

## Introduction

The importance of the spatial configuration of habitat patches and local environmental variables on the composition of communities, from microbes to trees, has been studied in a number of ecosystems, among them fresh waters and soils (Cottenie 2005; Nielsen et al. 2012; Caruso et al. 2013; Liu et al. 2013; Viketoft 2013). There is an emerging view that both the environment (e.g. habitat quality and suitability for species) and the spatial configuration of habitat patches (e.g. distance between habitat patches and reachability for species) are important forces shaping local and regional community composition and diversity (Leibold et al. 2004; Cottenie 2005; Gonzalez 2009; Marteinsdóttir 2014).

Terrestrial ecosystems are heterogeneous at multiple spatial scales, and the factors influencing this heterogeneity differ between scales (Levin 1992; Ettema and Wardle 2002; Berg 2012). At small scales up to a few tens of metres, habitat conditions and species movement among patches together determine the community structure of small soil organisms (Berg 2012; Martins da Silva 2012). The importance of environmental heterogeneity at small spatial scales is acknowledged, but its impact on species distribution has received less attention than spatial patterns at the scale of landscapes or beyond (e.g. Gaston 2000; Ettema and Wardle 2002; Zaitsev et al. 2013). The few spatially explicit studies on the small-scale distribution of soil organisms indicate that microbes (Saetre 1999), nematodes (Ettema et al. 2000; Viketoft 2013), microarthropods (Nielsen et al. 2010; Martins da Silva et al. 2012; Gao et al. 2014) and earthworms (Jimenez et al. 2006) often have an aggregated distribution. General conclusions of what actually causes spatial heterogeneity in species distribution are difficult to draw, as species differences in general life history, body size and home range can all have an effect (Berg 2012).

Structuring of communities can be linked to either environmental constraints on species, dispersal limitation, or biotic interactions (Vellend 2010). Partitioning variation in local community composition into environmental and spatial components is believed to provide information on important structuring processes (Borcard et al. 1992; Cottenie 2005). Variation predicted by only spatial variables is considered to reflect community dynamics that are not influenced by environmental constraints but by distance between habitat patches, while the joint contribution of both spatial and environmental variables could be either factors affecting both environmental variables and community structure simultaneously or the direct effect of environmental variables that are spatially structured. Several methods to incorporate space in the analysis of community structure have been developed (e.g. Borcard et al. 1992; Dray et al. 2006; Griffith and Peres-Neto 2006; Gilbert and Bennett 2010), with principal coordinates of neighbour matrices (PCNM) (Borcard and Legendre 2002; Borcard et al. 2004) being one of the most commonly used today. Combined with a trait-based analysis, variation partitioning may also suggest which characteristics of species are important in structuring these communities (Eros et al. 2009; Martins da Silva et al. 2012), and provide a mechanistic understanding of why environment or space structures communities.

An increasing body of research has indicated that species traits, rather than species identity, determines the responses of a species to environmental changes (de Bello et al. 2010; Cadotte et al. 2011; Lavorel et al. 2011). It has been argued that trait-based approaches can provide us with a better understanding of the important factors behind spatiotemporal shifts in community composition (Lavorel and Garnier 2002; McGill et al. 2006; Dias et al. 2013). For example, the turnover of species traits between communities across space is often smaller than the species turnover (Ackerly and Cornwell 2007; de Bello et al. 2009; Astor et al. 2014), indicating that species are replaced by other species with similar functional traits, if the environmental conditions of patches are similar. Trait-based approaches hold the promise of increasing the amount of variation explained when analysing organism–environment relationships. Whether trait-based approaches will help us to better understand organism–environment–space relationships at very small spatial scales too remains to be seen.

The aim of our study was to investigate the relative roles of environmental and spatial variables in small-scale patterns in the Collembola distribution of a salt marsh, using a trait-based approach. Collembola are a highly diverse, taxonomically and ecologically well-known taxon, and a dominant group of soil arthropods (Petersen and Luxton 1982; Hopkin 1997; Rusek 1998; Filser 2002). They often show significant changes in species composition across environmental gradients (Berg et al. 1998; Ponge and Salmon 2013). Recently, several studies have shown that Collembola traits are very useful for understanding shifts in community composition when environmental conditions such as temperature (Krab et al. 2010; Bokhorst et al. 2012), soil moisture (Makkonen et al. 2011), inundation (Russell and Griegel 2006), resource availability (Malmstrom 2012) or the level of habitat fragmentation (Martins da Silva et al. 2012) change.

Based on previous findings, we hypothesise that environmental variables are likely to be more important than the spatial configuration of habitat patches for Collembola community composition. If this hypothesis holds, then it follows that local communities consist of species that are more similar in traits to each other than expected by chance due to environmental filtering and, if there is variation in environmental factors across space, trait turnover between Collembola communities will be larger than expected by chance. To test these hypotheses, we investigated the distribution of Collembola in a salt marsh, where we used an extensive and spatially explicit sampling design on a scale of 25 m × 35 m. On this scale, the topography of the salt marsh is heterogeneous and the soil is regularly flooded with salt water, both of which are factors that affect most of the organisms inhabiting salt marshes.

## Methods

### Field site

We studied the spatial distribution of salt marsh Collembola on the barrier island of Schiermonnikoog, the Netherlands, between February and April 2011. The yearly temperature on the island is 10.2 ± 0.72 °C (mean ± SD), and rainfall is 824 ± 149.1 mm (Royal Dutch Meteorological Society, http://www.knmi.nl). Our study area was located at the oldest part of the salt marsh, a ~120-year-old grassland (53°28′57 N, 6°13′13 E) (Olff et al. 1997). The study plot was laid out between two large creeks, with an average elevation of 1.65 m above mean high tide (MHT). These creeks regularly overflow with salt water, more often between mid-September and mid-March, resulting in an average annual inundation frequency of 60 times year^−1^. The late-succession vegetation stage was dominated by the halophytic tall grass *Elytrigia atherica* (Link) Kerguélen (sea couch), and a sparse cover of *Juncus maritimus* Lam. (sea rush) tussocks was present (Schrama et al. 2012) (see Fig. ESM1 of the Electronic supplementary material, ESM). The site was selected because of the dynamic environmental conditions and heterogeneity in habitat factors that are known to affect Collembola, i.e. topography, thickness of the litter layer (resources) and vegetation height (shelter from harsh climatic conditions, especially heat waves and drought spells).

### Study organisms and sampling

Collembolan communities were sampled in April 2011. After assigning the sampling points (see below), the study plot was left undisturbed for five days before sampling, to minimize the effect of disturbance on species. Soil cores (diameter 10 cm, height 5 cm including a thin litter layer) were taken using a soil corer, carefully placed in plastic containers (diameter 11 cm), and transported to the field station (stored cool ~ 12 °C). After two days, the cores were transported to the VU University, Amsterdam, where Collembola were extracted using Tullgren extractors (following van Straalen and Rijninks 1982). Before extraction, the fresh weight of the soil cores was measured (to the nearest mg with a Sartorius balance). After three weeks of extraction, Collembola were identified to species using the identification keys of Fjellberg (1998, 2007) and Hopkin (2007) and counted.

### Spatially explicit sampling design

To establish the spatial distribution of Collembola across the plot, we used a spatially explicit sampling design (following the nested survey of Webster and Boag 1992). We created a plot, 35 m by 25 m, with a grid of 12 basal nodes. From these nodes, two series of seven and eight additional samples were assigned, respectively, with the distance between the centres of subsequent sampling points for each series decreasing from 3.2 m (in one of the series) to 1.6, 1.0, 0.8, 0.6, 0.4, 0.2 and 0 m (immediately adjacent to the previous sample). The node was included as a sample point, resulting in 16 samples per node. Each subsequent sample point was positioned away from the previous sample point in a randomly drawn compass direction (to the nearest grade). This resulted in a total of 192 sampling points (12 nodes × 16 samples per node). An additional 23 sample points were assigned to cover less sampled areas between the nodes. The sampling points were geo-referenced (to the nearest cm) using one of the plot corners as a reference point. The distances between all sampling points were calculated using basic geometry. During handling, 40 of the total of 215 samples were lost before identifying the Collembola, and another 3 samples were excluded as they were identified as clear outliers based on visual inspection of the data on moisture content. The lost samples were equally distributed over the nodes and series and the remaining 172 samples were used in further analyses. See Fig. ESM2 of the ESM for a schematic map and details of the sampling design.

### Environmental data

To understand the spatial distribution of Collembola species, we collected field data on abiotic and biotic variables at each sample point, viz. small-scale topography, soil moisture content, thickness of the litter layer and number of *J. maritimus* stems. Spatial differences in topography can influence the horizontal distribution of Collembola as they affect soil moisture content and vegetation patterning. Elevation (topography) determines soil moisture and frequency of flooding with salt water. We measured the height of the sampling points relative to the height of a theodolite (Nestle, type Nestor 6), as a proxy for topography. A theodolite was placed next to the base point (0, 0 coordinate) of the plot. It was not possible to calculate the actual height above MHT of the samples due to the absence of a reference point of known height above MHT in close proximity to the plot. Height (to the nearest cm) was measured by placing the measuring pole 1 cm to the north of the edge of the sample hole.

Presence of litter positively influences Collembola abundance as it delivers resources, offers protection against drought, and buffers fluctuations in air temperature. The thickness of the litter layer was measured with a ruler (to the nearest mm) on the south and north insides of the sample points after the soil cores were taken, and averaged per sample point.

Tall tussocks of *J. maritimus* provide shelter to Collembola, especially under extreme climatic events. The number of belowground stems is a proxy for the size of tussocks. After taking a soil core, the stems were clearly visible at the cutting edge of the soil sample (at 5 cm depth in the soil), and the numbers of alive and dead stems were counted.

Vegetation height determines the amount of aboveground litter, shades the soil from direct sunlight, and buffers against temperature and moisture fluctuations. A 1-m-long metal pole (diameter 5 mm) was placed at the centre of each sample point prior to soil core sampling. A plastic sliding disc (diameter 10 cm, 2 mm thick, with a 6-mm-diameter hole in the centre, 160 g) that ran down the pole until stopped by resistance from the vegetation was used to measure the height of the vegetation (to the nearest mm).

Soil moisture content (in %), litter mass (in g dry weight) and mass of remaining soil (in g dry weight) were calculated from the measurements of fresh weight and dry weight of the samples after soil fauna extraction (for details, see the section “Study organisms and sampling”). Soil cores were dried for 24 h at 50 °C.

### Collembolan trait data

To understand the variation in abundance and spatial distribution of Collembola species across the plot, we selected five traits that have previously explained shifts in collembolan species composition (Krab et al. 2010; Makkonen et al. 2011; Bokhorst et al. 2012; Martins da Silva et al. 2012; Van Dooremalen et al. 2013). The selected traits were: body size, antenna length to body length ratio, life form, moisture preference and habitat width (see Table 1 for definitions and rationale). Trait values (Table ESM1 in the ESM) were obtained from a large Collembola trait database (M. P. Berg, unpublished data). We did not measure any traits, so it was only possible to analyse the between-species (not within-species) variation in trait values.

**Table 1:** Species traits used in the analyses, their definitions and rationale, and the range of values of each trait for the species observed in samples

Trait	Definition	Ecological rationale	Range of values or categories
Body length	Maximum length from head to tip of abdomen (in mm)	Connected to dispersal ability, life form, ecophysiology^a, b^	0.5–5.4
Antennal to body length ratio	Antenna length divided by body length	Assumed to be linked to “sensory ability” and active dispersal^c^	0.1–0.7
Life form	Trait complex composed of number of ommatidia, length of springtail, and intensity of coloration^d^	Proxy for vertical stratification, ecophysiology and dispersal ability^b^	Epigeic (1)* Hemiedaphic (0.5) Euedaphic (0)
Moisture preference	Level of soil moisture content the species is mostly associated with^e^	Ability to tolerate high or low soil moisture contents^f^	Xerophile (0) Xero-mesophile (0.25) Mesophile (0.5) Meso-hygrophile (0.75) Hygrophile (1)
Habitat width	Number of habitat types in which the species has been found	Tolerance to environmental fluctuations, identifies generalists and specialists	1–9

* Epigeic surface-dwelling,* hemiedaphic* litter-dwelling,* euedaphic* soil-dwelling

References: ^a^Berg et al. (1998); ^b^ Ponge et al.( 2006); ^c^ Martins da Silva et al. (2012); ^d^ Gisin (1943); ^e^ Kuznetsova (2003); ^f^ Makkonen et al. (2011)

### Statistics

#### General

We excluded all environmental variables that were highly correlated (Pearson *r* > 0.6) from further analysis. Some variables were still correlated but to a low degree (Table ESM2 of the ESM). Variables that did not show normality were ln-transformed (i.e. vegetation height, litter thickness and litter mass), or square-root-transformed when they consisted of many zero values or were percentages (i.e. soil moisture content and number of *Juncus* stems).

To ensure equal weights of species traits in multi-trait analyses, we scaled the values of each trait between 0 and 1 (see Table 1). Pearson correlation tests showed that life form was positively correlated with body length and antenna length to body length ratio (Table ESM3 of the ESM). We nevertheless decided to keep all three traits for further analyses, as the correlations were weak (Pearson *r* = 0.50 and 0.55, respectively, Table ESM3 of the ESM).

The spatial configuration of samples was described by PCNM, a method based on computing the principal coordinates of a matrix of geographic neighbours (after Borcard et al. 2011), using ‘spantree’ in the R package vegan (Oksanen et al. 2015) and the package PCNM (Legendre et al. 2012) in R version 3.1.2 (R Core Team 2014). PCNM analyses are considered robust and suitable for discriminating between spatial and environmental effects on community composition (Griffith and Peros-Neto 2006). However, since PCNM has been criticised for giving inflated R values and overestimating the impact of space (Gilbert and Bennet 2010), we alternatively also performed multivariate trend surface analyses (Borcard et al. 1992). As this method gave similar results (but with the environmental component explaining more of the variation; Online Resource 2 of the ESM), we present the PCNM results below.

#### Partitioning of species and trait diversity

To examine if there was a higher species turnover than trait turnover between samples, we used additive partitioning of species and trait diversity measures, as suggested by de Bello et al. (2009, 2010). This was done to assess the proportion of *within community* (*alpha*) diversity and *among communities* (*beta* = turnover) diversity to *total regional* (*gamma*) diversity. We used the Simpson diversity index (hereafter referred to as “Simpson”) to describe species diversity, since it is easily comparable with Rao’s quadratic entropy (hereafter referred to as “Rao”), which describes the community functional diversity as the extent of dissimilarity in trait values among species in a community (de Bello et al. 2009). For each of the five species for which traits considered, we calculated the Rao index at each sampling point (α-Rao) using species abundance data, the abundance-weighted Rao index of the whole sampled area (γ-Rao), and the turnover between communities (β-Rao) (de Bello et al. 2010). By summing the extent of dissimilarity for all five traits, a multi-trait Rao describing the functional diversity of the community (Botta-Dukat 2005) was calculated and partitioned into α, β and γ components. We used the dbFD function in the FD package (Laliberté and Shipley 2013) in R version 3.1.1 (R Core Team^ 2014^) to calculate Simpson and Rao indices (Laliberté and Legendre 2010). Rao was calculated according to Botta-Dukat (2005). To avoid problems with the dependence of beta diversity on the level of alpha diversity, we used the Jost correction on all diversity values (Jost 2007).

#### Underdispersion versus overdispersion: environmental filtering or biological interactions

We tested whether local communities consisted of species more similar (called *underdispersed*) or dissimilar (*overdispersed*) to each other than expected by chance, and if trait turnover between local communities was smaller or larger than expected by chance. An observed β-Rao value that is lower than expected indicates a low turnover of traits (de Bello et al. 2009). Null models were created by using the full set of species observed in the study as the total species pool and randomising the identity and thereby trait values for each community while keeping abundance distribution and species richness within each community as observed (de Bello et al. 2009; Mason et al. 2012). Within-community (α) and turnover (β) Rao values were calculated as described under “Partitioning of species and traits diversity” for both the observed community and the null models. We tested whether the observed communities differed from the null models using one-sided permutation tests from the ade4 package in R (Dray and Dufour 2007) with the function “as.randtest” and 499 replicates (0.05 significance level). Analyses were performed on each of the five traits separately and on the multi-trait-Rao index.

#### Spatial and environmental variables structuring species and trait composition

To test if more of the variation in community composition was explained by environmental variables than by spatial configuration of samples, we performed multivariate analyses of the variation in species or trait composition between samples, and used variation partitioning to separate the effects of the two sets of variables. We calculated the community weighted mean (CWM) trait values for each of the five traits using the method of Garnier et al. (2004), weighing species traits in each sample by the relative abundance of the species. Initial detrended correspondence analyses (DCA) of the two response matrices—the species abundances and the CWM matrices, respectively—showed a short gradient length (species composition: 1.840, CWM trait: 0.761), indicating weak unimodality in the data (ter Braak and Smilauer 2002). Therefore, we used linear model redundancy analyses (RDA) for further analyses. RDA of the two datasets (species abundances or CWM trait composition) was performed using the function ‘varpart’ in the R package vegan (Oksanen et al. 2015) in R version 3.1.2. Redundancy analysis of CWM is considered a suitable method when examining trait–environment relationships at the community level (Kleyer et al. 2012). Species abundances were Hellinger-transformed before analyses, which is a good way of handling community data with many zeros without weighting rare species too highly (Legendre and Gallagher 2001). We used forward selection (permutation of residuals under a reduced model; stopping criteria: alpha >0.05; 999 permutations) on both spatial and environmental variables to select the variables that contributed most to each model. This minimizes the risk of co-linearity and enables us to include an equal number of variables from each group (in this case, five from each in both analyses). This makes it possible to compare the relative amounts of variance explained by the sets (Cottenie 2005). Datasets of the selected environmental and spatial variables and any combinations of these were constructed to perform separate RDAs from which the variance explanation of each part could be calculated using the sum of all canonical eigenvalues (Borcard et al. 1992). To evaluate the significance of models, permutation tests (999 permutations, pseudo-*F* statistics) were performed on all separate models (pure environment, pure spatial and full model with both sets) using anova.cca from the R package vegan (Oksanen et al. 2015).

#### Environmental variables affecting the community weighted mean traits

To test the relative predictability of environment and space for CWM traits, we included both as predictor variables in multiple linear regressions. Variables were centred in order to compare the regression results (Schielzeth 2010). Forward selection analyses were performed (using the R package packfor) to select explanatory variables; between three and five environmental variables were selected in the final models, and the same number of spatial (PCNM) variables (order of inclusion from forward selection). The residuals were normally distributed according to visual inspection and Shapiro–Wilk normality tests. The amount of variability explained by each set of predictor variables was assessed based on sum of squares decomposition and compared with the residual from each regression (Legendre and Legendre 1998).

#### Visualising environmental patterns and species/trait composition

To determine and visualise the distances at which environmental variables were autocorrelated, we created semi-variograms and kriging maps (Klironomos et al. 1999). Empirical semi-variograms were constructed using the GeostatisticalWizard in ArcGIS (ESRI, ArcGIS 10.0). We used the general “rule of thumb” (ESRI 2014) that only half of the maximum distance sampled should be used. We used NearestNeighbour statistics (SpatialAnalyst, ArcGIS) and corrected for the clustered configuration of samples in order to determine the appropriate lag size (i.e. the number of pairwise comparisons to be included in each distance category). We then adjusted the number of lags used to get a fitted semi-variogram that reached 15 m (max distance between two samples ~30 m). We calculated exponential, spherical and Gaussian semi-variograms and used cross-validation to select the one that best describes the data. After a model of the semi-variogram had been selected, kriging maps were drawn with the 4-section method and using the 5 nearest neighbours (Geostatistical Wizard, ArcGIS).

To visualise how trait and species composition relate to the spatial configuration of the environmental variables, we plotted the RDA scores of the first axes of the full models (environmental and spatial variables together) onto the kriging maps. This was done separately for CWM RDA scores and species RDA scores. As RDA scores go from negative to positive values, we rescaled them to obtain a good visual representation.

## Results

A total of 33,492 collembolans belonging to 20 species were collected and identified. The species list, the frequency of occurrence in the samples and the mean density and biomass of the species can be found in Table ESM4 in the ESM. Coleman rarefaction analysis showed that the sampling was extensive enough to cover the full diversity at the study site: after 50 % of the total of 172 samples, more than 90 % of the species had been found (data not shown).

### Species turnover versus trait turnover

When partitioning the total γ-diversity into α-diversity and β-diversity components, the turnover of species between samples (distance between samples 0–30 m) was much larger than the turnover of the five single traits analysed. While the species diversity was partitioned into almost equal proportions of α-diversity and β-diversity, a maximum of only 16 % of the diversity could be related to β-diversity for single traits and for the multi-trait-Rao (Table 2).

**Table 2:** Regional (γ-diversity) species and trait (combined and single) diversity

	γ-Diversity	% α	% β
Species diversity	5.80	50	50
Multi-trait diversity	1.87	84	16
Body length	1.26	94	6
Antenna/body length ratio	1.26	96	4
Life form	1.55	85	15
Moisture preference	1.44	94	6
Habitat width	1.35	94	6

% α and β give the proportional contributions of the local (α-diversity) and between-site turnover (β-diversity) components to the γ-diversity

Species diversity was calculated using the Simpson diversity index, trait diversity using Rao Q, and multi-trait diversity using all five traits together (see text for details). β-Diversity was calculated by additive partitioning

### Trait underdispersion versus overdispersion

**Fig. 1:**
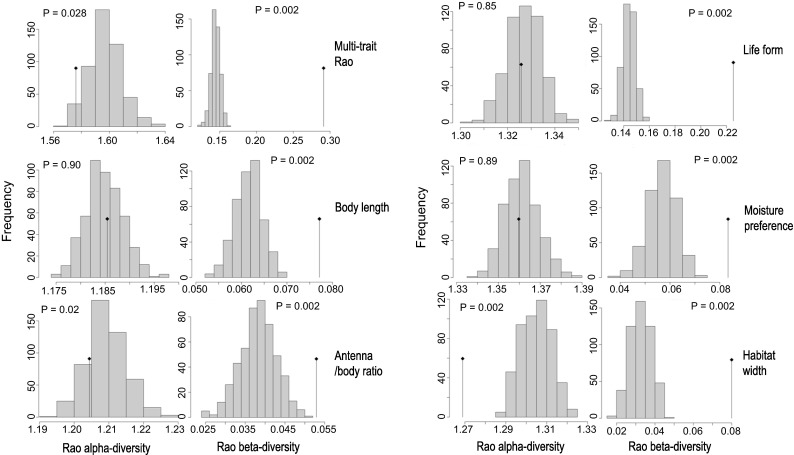
Partitioning of trait diversity values for all traits together (multi-trait-Rao) and for each single trait separately. The *bars* show the expected distributions of the trait values based on the observed species distributions (null model) for mean α-diversity and β-diversity, while the *flagpoles* show the corresponding observed values

For most traits, α-trait diversity did not differ significantly from what was expected by chance, i.e. neither trait underdispersion nor trait overdispersion was detected (Fig. 1). There were shifts in CWM trait values between samples, causing β-trait diversity to be significantly larger than expected (Fig. 1). Habitat width was the only trait that showed a lower than expected α-diversity and a higher than expected β-diversity. This indicates that, across samples, species differ considerably in habitat width, while species within a sample are more similar in this trait (i.e. they are either habitat generalists or specialists). Taking all five traits together (multi-trait-Rao), we found strong evidence for trait underdispersion, with lower α-diversity and higher β-diversity values than expected from a random distribution (Fig. 1). This was not found for any of the single traits except habitat width, suggesting that this trait is driving the multi-trait pattern.

### Effect of environmental variables and spatial location on species and trait composition

Spatially structured environmental variables (S∩E) explained the largest fraction of both species and trait composition in Collembola communities (species: 20.4 %, traits: 20.6 %). Pure environmental (E|S) and pure spatial (S|E) variables (5 variables together from each group) each explained low but significant proportions of variation in community composition (species: E|S = 13.6 %, S|E = 6.5 %, traits: E|S = 15.2 %, S|E = 7.4 %), with the environmental variables explaining marginally more. The environmental variable that contributed most to the canonical RDA axes was site topography. This variable alone explained more than half of the variation explained by all environmental variables together, i.e. 22 % for species identity and 19.5 % for species traits. The amount of unexplained variation was similar between the two ways of describing community composition (species: 59.5 %, traits: 56.8 %).

### Environmental variables affecting community weighted mean trait values

For all CWM trait values when they were analysed separately, variation across samples was related to changes in environmental variables. There was no single dominating environmental gradient along which shifts in CWM trait values occurred, as several environmental variables affected the majority of the CWM trait values (Table 3). Most species traits were affected by combinations of site topography, soil moisture and litter layer thickness (variables that are to some degree correlated; see Table ESM2 in the ESM). Vegetation height, which was not correlated with any other environmental variable measured, also seemed to be important. When we analysed the effect of each environmental variable on CWM traits separately, most of the models were significant but had low explanatory power (most *R*^2^ values <0.15; Table ESM5 in the ESM).

**Table 3:** Summary of multiple linear regressions between environmental and spatial variables, and community weighted mean traits

Trait	Significant variables including direction of correlation		Adj *R* ^2^
	Environmental	Spatial	
Body length***	Soil moisture (−)	V8 (−)	0.506
	Vegetation height (−)	V2 (−)	
	Litter thickness (−)	V12 (−)	
	(Litter mass) (−)	V16 (−)	
	(Topography) (+)	V3 (+)	
Antenna/body length ratio***	Vegetation height (−)	V8 (−)	0.464
	Soil moisture (−)	V2 (−)	
	(Litter mass) (−)	V1 (−)	
	(Topography) (−)	V11 (−)	
Life form***	Soil moisture (−)	V2 (−)	0.591
	(Topography) (+)	V8 (−)	
	Litter thickness (−)	V3 (+)	
	Vegetation height (−)	V16 (−)	
	(Litter mass) (−)	V1 (−)	
Moisture preference***	Vegetation height (−)	V1 (+)	0.239
	Topography (−)	(V15) (−)	
Habitat width***	Litter thickness (+)	(V3) (+)	0.317
	Topography (+)	(V8) (−)	
	Soil moisture (−)	V16 (−)	

Variables are shown in order of amount of variance explained based on an analysis of variance table. Variables that do not contribute significantly (*P* > 0.05) to the final model are shown in brackets. The direction of the relationship (positive or negative) is given after each variable. *** *P* < 0.001

**Fig. 2:**
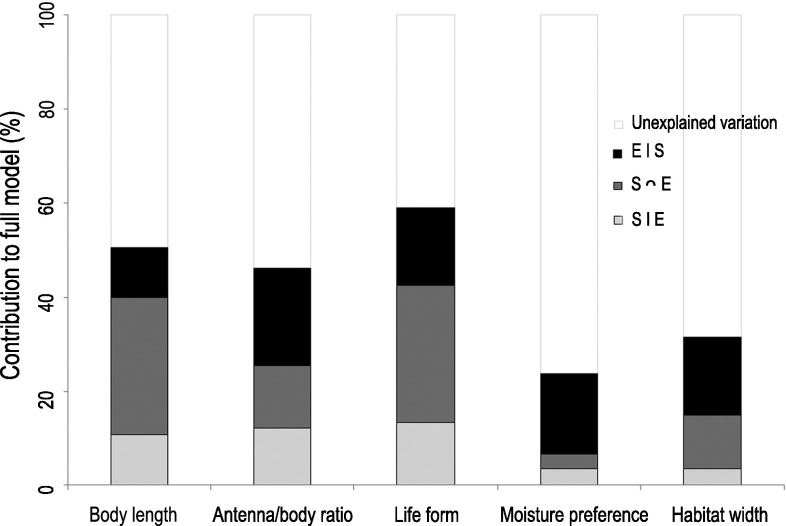
Proportions of the variance in the single trait community weighted mean explained by pure spatial (S|E), joint spatial and environmental (S∩E), and pure environmental (E|S) variables, respectively. Explanatory variables are included; *R* ^2^ values and the significance of each model are shown in Table 3

Spatially structured environmental variables or pure environmental variables usually explained more of the variation in each CWM trait across samples than pure spatial variables (Fig. 2). This suggests that the environmental variation is to some degree spatially structured and that this affect the CWMs. The levels of variation that could be explained by the full set of spatial and environmental variables were relatively high (*R*^2^ values 0.24–0.59, Fig. 2; Table 3).

### Spatial component of environmental variables

**Fig. 3:**
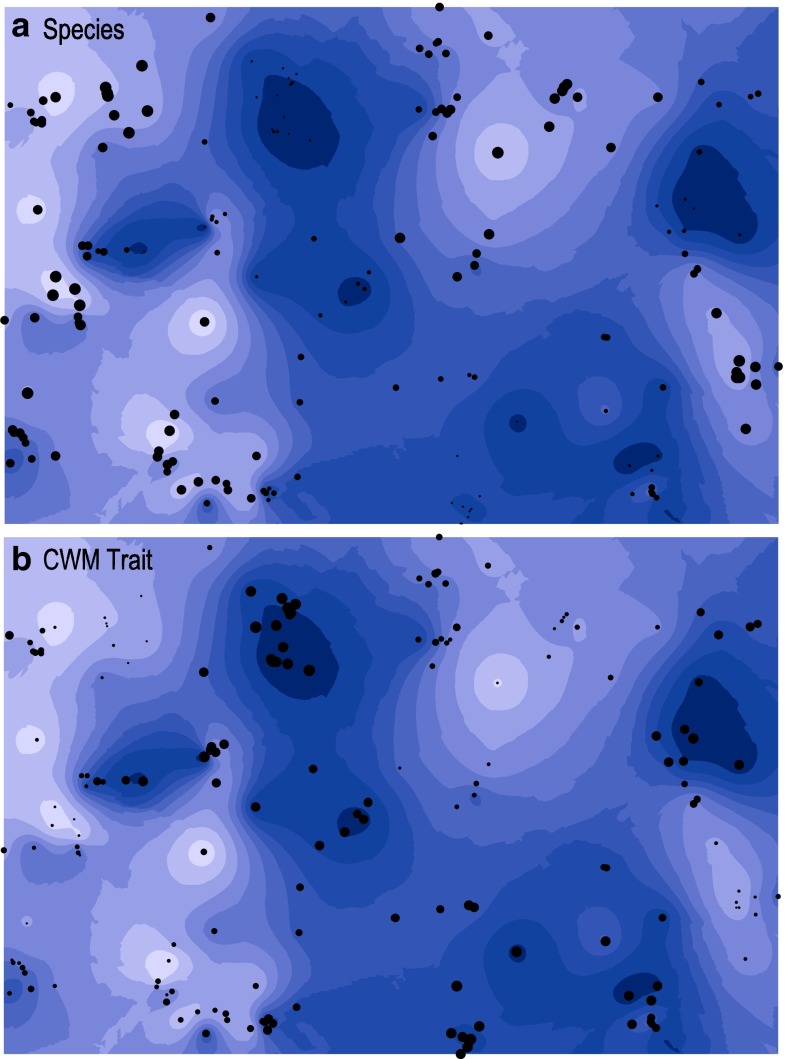
Interpolated topography profile of the study plot (*dark* low elevation points, *light* high elevation points) and RDA scores for **a** collembolan species composition and **b** CWM trait composition. *Small and large dots* represent low and high values on the first axis in the RDA (5 environmental and 5 spatial variables included) for species composition (eigenvalue = 0.103) and trait (CWM) composition (eigenvalue = 0.025)

We found that topography and soil moisture were autocorrelated within a distance of 4-6 meters (see Fig. ESM3 and Table ESM6 in the ESM). The variables describing species community variation, i.e. RDA scores, had a similar spatial range of ~5 m; however, a spatial autocorrelation of ~9 m was observed for CWM trait composition. The spatial scale of autocorrelation for the remaining environmental variables (litter thickness, litter mass, vegetation height, and number of stems) and the total abundance of Collembola could not be successfully estimated by any of the semi-variogram models based on cross-validation. Kriging maps, which are used to extrapolate values of variables in-between sampling points, show that the spatial variation in topography relates to the RDA scores describing community composition from variance partitioning (Fig. 3), i.e. for CWM trait composition (Fig. 3b), high RDA scores are more common at high elevation while low RDA scores are mainly found in low-elevation areas, and the reverse relation is true for species composition.

## Discussion

In the studied salt marsh, environmental conditions—in particular site topography and soil moisture—were the most important determinants of small-scale spatial variation in collembolan community structure. These environmental variables were spatially structured and, accordingly, we found that the spatially structured environment explained the largest proportion of the explained variation in trait as well as species composition. Turnover of species was high and turnover of  all traits combined was larger than expected by chance, supporting the notion that environmental variation across space is important for collembolan community composition.

On the small spatial scale studied, species diversity turnover was larger than trait diversity turnover. This was true for all five single traits analysed, as well as for the traits combined. Lower turnovers of traits than species have been shown for other groups of organisms analysed over larger spatial scales, mainly plants (Ackerly and Cornwell 2007; de Bello et al. 2009; Leps et al. 2011), but also land snails (Astor et al. 2014). Our findings suggest that on small spatial scales, species that are absent from a local community may be replaced by other species with similar trait values. Whether this indicates strong local competition between species with similar traits or rapid colonisation of empty niches by species with equivalent realised niches from surrounding habitat patches with similar environmental conditions requires further studies. Furthermore, although we have not measured the effects of shifts in species composition on ecosystem functioning, these results suggest that differences in soil processes between areas may be smaller than the turnover of species in this system.

The small spatial scale that we worked on (0–30 m) has rarely been used in studies of the mechanisms behind soil arthropod community composition (but see e.g. Berg and Bengtsson 2007; Berg 2012; Gao et al. 2014). Most Collembola species have small home ranges, even though they can disperse over longer distances, both actively and with the aid of winds or water flow (Freeman 1952; Hågvar 2000; Moore 2002; Hawes et al. 2008). Using sampling distances of <1 m to a few metres is therefore an appropriate spatial scale when examining mechanisms that decide local community composition. For several of the measured environmental variables, the range of autocorrelation (indicating the spatial scale on which they are not independent) was within a few metres, similar to (or a bit smaller than) the autocorrelation of collembolan community composition. We suggest that this small scale might be of great ecological importance in these communities and should therefore be given more attention in further community and population studies.

Our hypothesis that environmental variables were more important than the pure spatial configuration of habitat patches for both species and trait composition was largely confirmed, with small-scale topography being an important factor. Gilbert and Bennett (2010) have shown that ordination models often underestimate the environmental component compared to the spatial one. It is therefore possible that the importance of environmental factors for community composition is even larger than our results show. This problem of underestimation is often smaller when using the trend surface method (Borcard et al. 1992) instead of the PCNM method to describe space (Gilbert and Bennett 2010). We applied this alternative method and found that the spatial component explained an even smaller part of the variation in both species and trait composition (see Tables ESM1 and ESM2 in the ESM). These results indicate that, at least in salt marshes with distinct environmental gradients induced by differences in elevation, environmental factors are more important in driving local Collembola community composition than pure spatial factors.

Some studies on Collembola community composition, ranging from landscape (km) to plot (m) scale, show that community composition is better explained by environmental than spatial variables (Martins da Silva et al. 2012; Ponge and Salmon 2013). Other studies on soil fauna have shown other results. For nematodes, the abiotic, biotic and spatial variables had similar influences on species distribution (Viketoft 2013), while spatial configuration was more important than environmental variables for oribatid and mesostigmatid mites (Lindo and Winchester 2009; Nielsen et al. 2012; Gao et al. 2014). Experimental studies have shown that oribatid mites are dispersal limited (at distances >5 cm), while collembolans did not show dispersal limitations of up to 3 metres (Åström and Bengtsson 2011).

In the present study using the PCNM method, 43 % of the variation in community trait composition across space was explained by spatial and environmental variables, compared to 41 % for species composition. The proportion of unexplained variance, more than 50 %, suggests that there are additional factors which were not accounted for here that have an effect on species composition. Environmental factors that were not measured in this study, such as small-scale variations in salinity (see below), local differences in the ground cover of *Elytrigia atherica* (sea couch), or differences in litter quantity and quality, are possible candidates.

We found that the CWM of each trait was influenced by a strong spatial heterogeneity in environmental factors. Soil moisture and litter thickness (both correlated with topography) were the most important environmental variables, together with vegetation height. The thickness of the litter layer determines the amount of suitable habitat available for many Collembola species and high vegetation shelter from direct sunlight, buffering against temperature and moisture fluctuations.

**Fig. 4:**
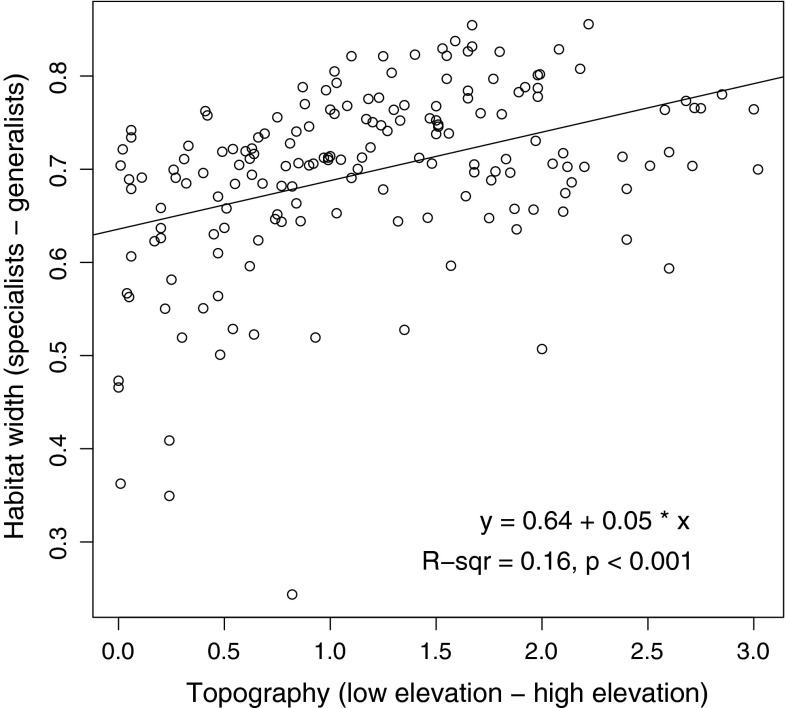
Community weighted mean habitat width as a function of plot topography. Average habitat width decreases as the elevation decreases, indicating the presence of more specialist species in depressions of the study plot

Local communities consisted of species that are more similar in traits than expected by chance. This is likely a consequence of a strong environmental filtering of species in our study area. Further support for this conclusion is provided by the fact that for all traits the turnover between local communities was larger than expected by chance. Habitat width showed lower α-diversity and a higher β-diversity than expected; the CWM of habitat width was best explained by litter thickness, topography and soil moisture, suggesting that these variables influence community composition. Habitat width is assumed to describe whether species are adapted to a narrow habitat niche or can tolerate a wide range of conditions. An examination of the relationship (Fig. 4) between CWM habitat width and site topography indicates that the low-elevation points have communities that mostly consist of habitat specialists. The low-elevation areas are likely to be more affected by salt-water flooding and probably experience higher salinity and longer periods of water logging (Bakker et al. 1985; Olff et al. 1997). These conditions are stressful for Collembola and would select for species that are adapted for these particular conditions. Unfortunately, we lack good data on the salinity tolerances of the species found in our study, so we cannot examine if species that are specialised to saline conditions occur more often at low elevation areas.

We believe that the patterns we see in trait distribution, with underdispersion in some traits and variation in trait composition explained to a large degree by environmental variables, are a result of the fluctuating environment and functional traits of individuals that enable them to cope with these fluctuations. In heterogeneous habitats, it is commonly found that the environmental filtering is stronger than the process of niche partitioning (Mason et al. 2013). Salmon et al. (2014) have previously shown that variations in traits of collembolan communities are linked to environmental conditions, and depending on the traits analysed they could explain 20–70 % of the variation in a broad-scale (cross-Europe) study.

Life form was the trait most related to local environmental and spatial variables (Table 3), and in trait diversity partitioning it showed the largest β-diversity component (Table 2). Life form is determined by the vertical position of the species in the soil and is correlated to both body size and sensory ability. Large-bodied surface-dwelling species have long antennae, while short-bodied soil-dwelling species have reduced antenna lengths (Gisin 1943). “Life form” therefore describes the microhabitat niche position, resource utilisation, and dispersal ability. It has previously been shown to be important to determine how collembolan communities respond to climate change (Makkonen et al. 2011) or crop selection on contaminated land (Chauvat et al. 2014) and recovery after fire (Malmström 2012). As it is quite easy to categorise collembolans into life forms, we suggest that life form is a good trait to use for further studies of community composition and functions of collembolans.

## Conclusions

We found that most of the variation in collembolan communities across space (0–30 m) was explained by spatial variation in small-scale topography. Topography determines environmental factors such as inundation that in turn probably influence the distribution of collembolan species. The communities in our study were mostly structured based on traits related to life form but also on disturbance tolerance, which was reflected in the higher proportion of habitat specialists in low-elevation areas. Our results suggest that if a species is lost from a community, a species with similar traits usually takes its place. We believe that trait approaches in ecological studies have the potential to improve our understanding of the processes leading to the assembly of communities.

### Author contribution statement

MB and JB originally formulated the idea; LAW, MB, JB and ÅB developed the theoretical framework further; MB, KZ and ES designed the study and conducted fieldwork; MB, FH-B, KZ and ES identified the species; MB and LAW summarised traits data for the database; LAW performed the statistical analyses; LAW, JB, MB and ÅB wrote the manuscript, with LAW doing the majority of the work.

## Electronic supplementary material

Supplementary material 1 (DOCX 737 kb)

Supplementary material 2 (DOCX 42 kb)
