# Supplementary material for: Spatially structured environmental filtering of collembolan traits in late successional salt marsh vegetation
Source: Oecologia. 2015 May 23;179(2):537–49. doi: 10.1007/s00442-015-3345-z (PMC4568007; doi:10.1007/s00442-015-3345-z)
Supplement: Supplementary file 1 — Supplementary material 1 (DOCX 737 kb) [file 442_2015_3345_MOESM1_ESM.docx]

**ELECTRONIC SUPPLEMENTARY MATERIAL (Online Resource 1)**

**Spatially structured environmental filtering of Collembola traits in late successional salt marsh vegetation**

Lina A. Widenfalk^1^, Jan Bengtsson^1^, Åsa Berggren^1^, Krista Zwiggelaar^2^, Evelien Spijkman^2^, Florrie Huyer-Brugman^2^ and Matty P. Berg^2, 3^

1. Department of Ecology, Swedish University of Agricultural Sciences, P.O. Box 7044, Uppsala SE-75007, Sweden
2. Department of Ecological Sciences, VU University, Amsterdam, De Boelelaan 1085, 1081 HV Amsterdam, The Netherland
3. Community and Conservation Ecology Group, Center for Ecological and Evolutionary Studies, University of Groningen, Nijenborgh 7, 9747 AG Groningen, The Netherlands

Corresponding author: Lina A. Widenfalk

e-mail: [lina.ahlback@slu.se](mailto:lina.ahlback@slu.se)

telephone: +46(0)18-67 20 21

Fax no: +46(0)18-672890


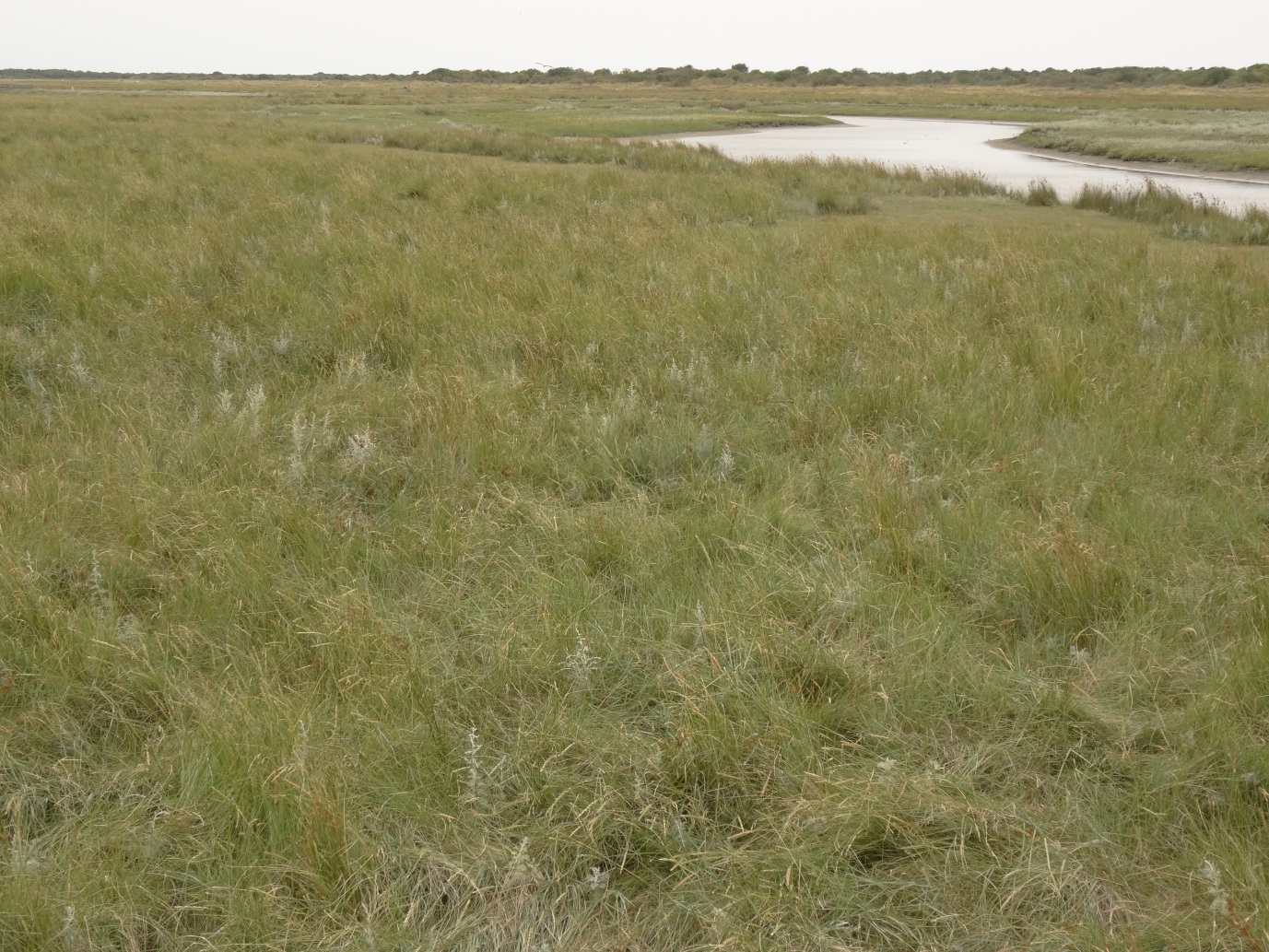


**Fig. ESM1** A view of the late successional vegetation stage (about 150 y old) of the salt marsh on the barrier island Schiermonnikoog. The vegetation looks homogeneous to the human eye but variation in topography and soil moisture content were observed, which affect the composition of local Collembolan communities. For further details see the main text. Photo M.P. Berg

To establish the spatial distribution of Collembola across the plot we used a spatially explicit sampling design (following the nested survey of Webster and Boag 1992). We created a plot, 35 m by 25 m, with a grid of 12 basal nodes, 3 rows of 4 nodes oriented along a North to South compass angle (Fig. ESM2A). Distance between the nodes was 7 m in the North to South direction and 6.25 m in the East to West direction, with the outermost nodes always at a distance of at least 5 m from the edge of the plot. At each node, two series of an unequal number of additional sample points (7 and 8 samples) were assigned, giving 16 samples per node as the node was also a sampling point. Distance between the centres of sampling points was 3.2 m, 1.6 m, 1.0 m, 0.8 m, 0.6 m, 0.4 m, 0.2 m and ~0 m (immediately adjacent), with the exception that the largest distance (3.2 m) was only included in one of the series for each node. The spatial positioning of the subsequent samples in the field was based on randomly selected compass angles. This design gave 192 sampling points (12 nodes × 16 samples per node). An additional 23 sample points were assigned, two to each node with a distance of 2.4 m to the node, to cover less sampled areas between the nodes. Unfortunately, during handling 40 of the total of 215 samples were lost before identification of the Collembola, and another 3 samples were excluded as they were identified as clear outliers based on visual inspection of data on moisture content. Fortunately, the lost samples were equally distributed over the nodes and series and the remaining 172 samples were used in further analyses (Fig. ESM2B).

a. full design

b. analysed samples

**Figure ESM2**. **A** schematic view of the study plot with sample points with A; the full design above and **B**, including only the samples used in analyses below. Middle points (black square) in each segment represent 12 grid nodes, separated by 7 m (x-direction) and 6.25 m (y-direction).

Diamond-shapes represent the subsequent sample points. Lines depict two Series of sampling points per node, connecting sample points at fixed distances of 3.2 m (only Series one), 1.6, 1, 0.8, 0.6, 0.4, 0.2, and ~0 m (immediately adjacent). In Series two the distances between subsequent samples are the same, but the distance from the first sample to the node is 1.6m. Different colours represent samples belonging to different grid nodes. Black squares not connected with any line indicate the additional sampled points.

*Reference:*

Webster R, Boag B (1992) Geostatistical analysis of cyst nematodes in soil. J Soil Sci 43:583-595

**Table ESM1** Species trait values used in the analyses. All traits where scaled between 0-1 to allow for multi-trait analyses.

| Species | Body  length | Antenna/body  ratio | Life  form | Moisture  preference | Habitat  width |
| --- | --- | --- | --- | --- | --- |
| *Arrhopalites caecus* (Tullberg, 1871) | 1.0 | 0.51 | eu | meso | 6 |
| *Brachystomella parvula* (Schaeffer, 1896) | 1.0 | 0.18 | hemi | meso | 4 |
| *Ceratophysella succinea* (Gisin, 1949) | 1.8 | 0.11 | hemi | hygro-meso | 9 |
| *Dicyrtomina minuta* (O. Fabricius, 1783) | 2.5 | 0.74 | epi | hygro | 5 |
| *Entomobrya lanuginosa* (Nicolet, 1841) | 2.0 | 0.52 | epi | xero | 4 |
| *Entomobrya nivalis* (Linnaeus, 1758) | 2.0 | 0.52 | epi | xero | 3 |
| *Folsomia sexoculata* (Tullberg, 1871) | 2.0 | 0.17 | hemi | meso | 1 |
| *Friesea mirabilis* (Tullberg, 1871) | 1.9 | 0.13 | hemi | meso-hygro | 8 |
| *Halisotoma maritima* (Tullberg, 1871) | 1.7 | 0.24 | hemi | meso-hygro | 2 |
| *Isotoma anglicana* Lubbock, 1862 | 3.6 | 0.24 | hemi | meso | 8 |
| *Isotoma riparia* (Nicolet, 1842) | 5.4 | 0.24 | epi | hygro | 5 |
| *Lepidocyrtus violaceus* (Geoffroy, 1762) | 1.5 | 0.38 | epi | xero-meso | 7 |
| *Mesaphorura macrochaeta* Rusek, 1976 | 0.7 | 0.09 | eu | hygro-meso-xero | 7 |
| *Neanura muscorum* (Templeton, 1835) | 3.5 | 0.16 | hemi | hygro-meso | 8 |
| *Parisotoma notabilis* (Schaeffer, 1896) | 1.0 | 0.28 | hemi | meso | 6 |
| *Sminthurinus aureus* (Lubbock, 1862) | 1.0 | 0.44 | hemi | hygro | 6 |
| *Sminthurus viridis*(Linnaeus, 1758) | 3.0 | 0.53 | epi | xero-meso | 2 |
| *Sphaeridia pumilis* (Krausbauer, 1898) | 0.5 | 0.43 | hemi | meso | 4 |
| *Thalassaphorura debilis* Moniez, 1889 | 1.4 | 0.11 | eu | hygro-meso | 1 |
| *Xenylla maritima* Tullberg, 1869 | 1.4 | 0.15 | hemi | xero | 6 |

**Body length** – length of body in mm; **Antenna/body ratio** – the ratio between antenna length and body length; **Life form** – vertical stratification, eu = Euedaphic – living in deeper soil layers, hemi = Hemiedaphic – living in the litter layer, epi = Epigaeic – living on the soil surface or in the vegetation; **Moisture preference** – general preference of moisture level, xero = Xerophile – lives in dry conditions, meso = Mesophile – lives in intermediate moisture conditions, hygro = Hygrophile – lives in wet conditions; **Habitat** **width** – no of habitat categories the species can be found in. All data obtained from a large Collembola trait database (Berg, unpublished data).

**Table ESM2** Correlations between environmental variables, after removing three outliers based on very high soil moisture contents.

|  | Veg. height | Stems | Litter  thickness | Topography | Litter mass | Soil mass | Moisture |
| --- | --- | --- | --- | --- | --- | --- | --- |
| Veg height | 1 | 0.065 | -0.033 | -0.060 | 0.021 | -0.014 | 0.026 |
| Stems | 0.065 | 1 | 0.185  * | -0.027 | 0.079 | -0.103 | 0.117 |
| Litter thickness | -0.033 | 0.185  * | 1 | -0.289  *** | 0.339  *** | -0.260  *** | 0.285  *** |
| Topography | -0.060 | -0.027 | -0.289  *** | 1 | -0.115 | 0.101 | -0.502  *** |
| Litter mass | 0.021 | 0.079 | 0.339  *** | -0.115 | 1 | -0.394  *** | 0.057 |
| Soil mass | -0.014 | -0.103 | -0.260  *** | 0.101 | -0.394  *** | 1 | -0.498  *** |
| Moisture | 0.026 | 0.117 | 0.285  *** | 0.502  *** | 0.057 | -0.498  *** | 1 |

*** P < 0.001, ** P < 0.01, * P < 0.05 Veg. height = Vegetation height (cm), Stems = Total nr. of *Juncus maritimus* stems at -5 cm in the soil, Litter thickness = Average thickness of the litter layer (cm), Topography = Relative height in comparison to a theodolite (cm), Litter mass= Dry mass litter layer (mg), Soil mass = Dry mass rest of soil (after removing litter) (mg), Moisture = Soil water content (%).

**Table ESM3** Pearson correlations between Collembola species traits. Traits are scaled between 0-1 to account for differences in expression. * P < 0.05

|  | Body length | Antenna/body ratio | Life form | Moisture preference | Habitat width |
| --- | --- | --- | --- | --- | --- |
| Body length | 1 |  |  |  |  |
| Antenna/body ratio | 0.013 | 1 |  |  |  |
| Life form | 0.495 * | 0.545 * | 1 |  |  |
| Moisture preference | 0.244 | -0.139 | -0.213 | 1 |  |
| Habitat width | 0.072 | -0.218 | -0.107 | 0.166 | 1 |
|  |  |  |  |  |  |

**Table ESM4** Collembola species found in the litter and soil of late successional vegetation of a salt marsh. The order of the species is based on the frequency of occurrence in the samples (n = 172).

| Species | Frequency in samples  (%) | Density per sample  mean (no) + sd | | Biomass per sample  mean (µg) + sd^a^ | |
| --- | --- | --- | --- | --- | --- |
| *Xenylla maritima* Tullberg, 1869 | 100 | 30.80 | 36.10 | 798 | 935 |
| *Isotoma anglicana* Lubbock, 1862 | 98 | 23.30 | 24.30 | 5194 | 5429 |
| *Friesea mirabilis* (Tullberg, 1871) | 97 | 19.20 | 19.50 | 586 | 596 |
| *Mesaphorura macrochaeta* Rusek, 1976 | 90 | 84.60 | 159.40 | 118 | 219 |
| *Dicyrtomina minuta* (O. Fabricius, 1783) | 88 | 5.60 | 6.04 | 8578 | 9248 |
| *Folsomia sexoculata* (Tullberg, 1871) | 81 | 19.80 | 29.10 | 802 | 1243 |
| *Isotoma riparia* (Nicolet, 1842) | 81 | 2.51 | 2.50 | 1585 | 1563 |
| *Lepidocyrtus violaceus* (Geoffroy, 1762) | 66 | 3.43 | 5.70 | 1235 | 2055 |
| *Sminthurus viridis*(Linnaeus, 1758) | 60 | 2.37 | 3.50 | 4473 | 6542 |
| *Sphaeridia pumilis* (Krausbauer, 1898) | 57 | 2.49 | 5.50 | 20 | 44 |
| *Entomobrya lanuginosa* (Nicolet, 1841) | 16 | 0.20 | 0.51 | 125 | 320 |
| *Halisotoma maritima* (Tullberg, 1871) | 14 | 0.64 | 5.74 | 24 | 216 |
| *Thalassaphorura debilis* Moniez, 1889 | 12 | 0.60 | 2.55 | 13 | 55 |
| *Brachystomella parvula* (Schaeffer, 1896) | 5 | 0.06 | 0.31 | 0.27 | 1.30 |
| *Ceratophysella succinea* (Gisin, 1949) | 3 | 0.04 | 0.27 | 1.05 | 7.06 |
| *Parisotoma notabilis* (Schaeffer, 1896) | 2 | 0.02 | 0.13 | 0.11 | 0.84 |
| *Arrhopalites caecus* (Tullberg, 1871) | 1 | 0.02 | 0.17 | 1.16 | 11.3 |
| *Entomobrya nivalis* (Linnaeus, 1758) | 1 | 0.01 | 0.08 | 6.16 | 80.8 |
| *Neanura muscorum* (Templeton, 1835) | 1 | 0.01 | 0.08 | 1.17 | 15.4 |
| *Sminthurinus aureus* (Lubbock, 1862) | 1 | 0.06 | 0.69 | 3.86 | 45.8 |

a, The biomass of each single species was estimated using body length-to-dry mass allometric relationships following Caballero et al. (2004) who gives relationships for four basic body forms in Collembola. Encountered species were allocated to one of the four basic groups and the body form specific allometric relationship was used to calculate species specific dry mass. Species sample biomass was calculated by multiplying the species-specific dry mass with the abundance in each sample.

*Reference:*

Caballero M, Baquero E, Arino AH, Jordana R (2004) Indirect biomass estimations in Collembola. Pedobiologia 48:551-557. doi: 10.1016/j.pedobi.2004.06.006

**Table ESM5** Relationship between community weighted mean traits and environmental variables, based on pairwise regressions between each trait and single environmental variables. Only environmental variables included in the final model of the analyses are shown, variables not showing significance in analyses of variance are within brackets.

| Traits | Env. variable | Estimate | Adj R^2^ | Sum Sq | F-value | Sign |
| --- | --- | --- | --- | --- | --- | --- |
| Body length | Topography | 0.061 | 0.195 | 0.365 | 42.75 | *** |
|  | Soil moisture | -0.013 | 0.242 | 0.315 | 34.94 | *** |
|  | Vegetation height | -0.047 | 0.052 | 0.105 | 10.39 | ** |
|  | Litter thickness | -0.078 | 0.073 | 0.143 | 14.56 | *** |
|  | Litter mass | -0.081 | 0.077 | 0.156 | 15.68 | *** |
| Antenna/body ratio | Topography | 0.043 | 0.149 | 0.179 | 31.22 | *** |
|  | Vegetation height | -0.056 | 0.124 | 0.150 | 25.37 | *** |
|  | Soil moisture | -0.009 | 0.157 | 0.188 | 33.00 | *** |
|  | Litter mass | -0.054 | 0.054 | 0.069 | 10.82 | ** |
| Life form | Topography | 0.123 | 0.305 | 1.457 | 76.54 | *** |
|  | Soil moisture | -0.022 | 0.270 | 1.293 | 64.72 | *** |
|  | Litter thickness | -0.182 | 0.165 | 0.802 | 35.10 | *** |
|  | Vegetation height | -0.051 | 0.021 | 0.125 | 4.67 | * |
|  | Litter mass | -0.108 | 0.052 | 0.271 | 10.44 | ** |
| Moisture preference | Vegetation height | -0.083 | 0.173 | 0.324 | 36.93 | *** |
|  | (Topography) | (-0.021) | (0.018) | (0.043) | (4.15) | (*) |
| Habitat width | Topography | 0.052 | 0.160 | 0.261 | 33.71 | *** |
|  | Litter thickness | 0.044 | 0.024 | 0.046 | 5.15 | * |
|  | (Soil moisture) | (-0.008) | (0.109) | (0.181) | (22.00) | (***) |


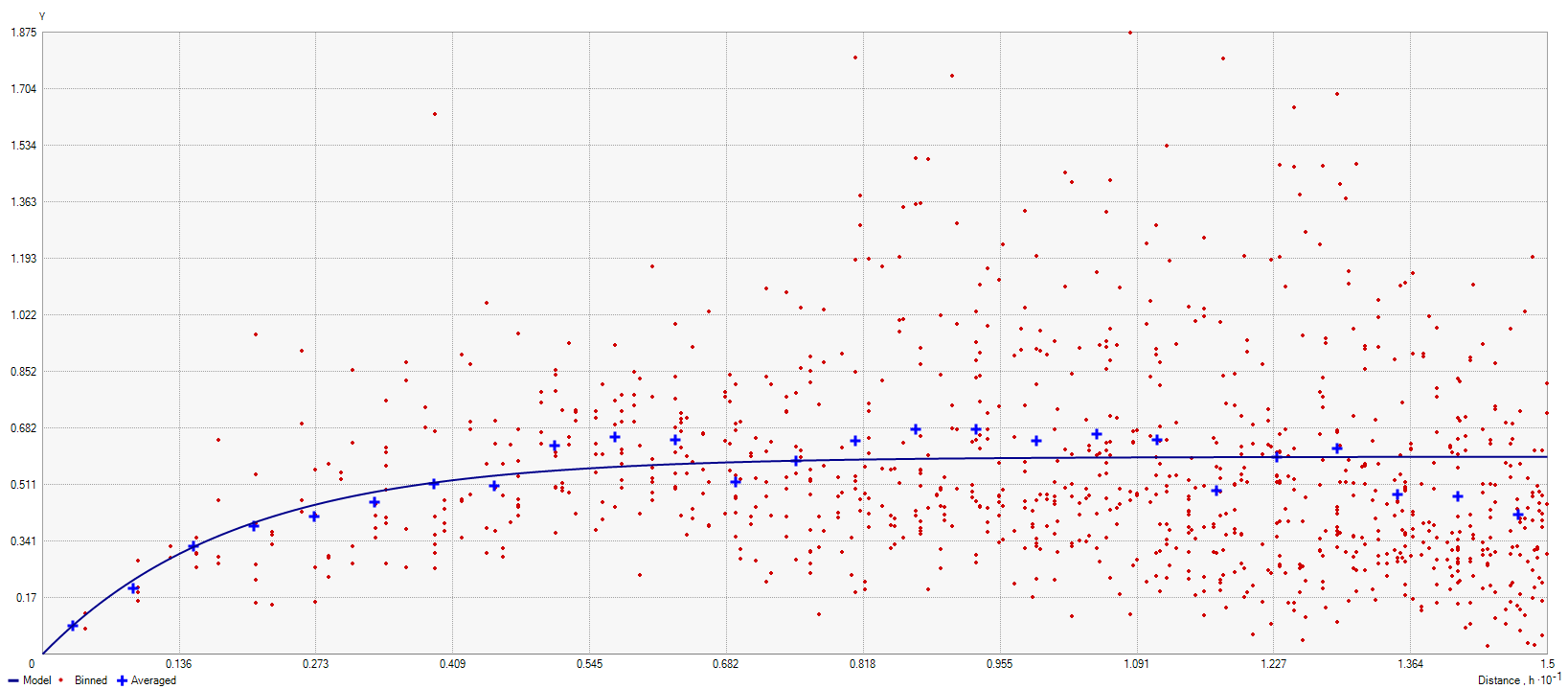
**Fig. ESM3** Semivariogram of topography, with the exponential model shown in Table ESM7

**Table ESM6** The model parameters used for the fitted semivariograms.

| Response variable | Model | Major range | Nugget | Partial sill |
| --- | --- | --- | --- | --- |
| Topography | **Exponential** | **5.760** | **0** | **0.595** |
|  | Spherical | 4.772 | 0.032 | 0.555 |
|  | Gaussian | 3.655 | 0.093 | 0.491 |
| Moisture% | **Exponential** | **4.242** | **0.011** | **0.058** |
|  | Spherical | 3.275 | 0.015 | 0.054 |
|  | Gaussian | 2.866 | 0.022 | 0.047 |
| *Vegetation height* | Exponential | 0.941 | 0 | 81.77 |
|  | Spherical | 0.436 | 0 | 0.279 |
| *Litter thickness* | Exponential | 7.763 | 0.105 | 0.038 |
| *Total no of stems* | Exponential | 0.416 | 0.746 | 1.805 |
|  | Spherical | 0.578 | 1.457 | 1.095 |
| *Litter mass* | Exponential | 12.923 | 0.010 | 0.043 |
| *Collembola abundance* | Exponential | 7.580 | 0.144 | 0.345 |
|  | Gaussian | 6.134 | 0.222 | 0.261 |
| RDAscore species | **Exponential** | **5.326** | **0.001** | **0.015** |
|  |  |  |  |  |
| RDAscore CWM | **Exponential** | **9.369** | **0** | **0.008** |
|  |  |  |  |  |

The models marked in bold font were considered giving the best fit based on cross-evaluation of predicted and observed values. Variables for which no model was considered to have a good fit are marked in italic. For all fitted semivariograms we consequently used a Lag size of 0.6 m (based on mean distance nearest neighbour) and no. of lags = 25, giving a distance of 15 m (half the measured distance).
