# Supplementary material for: Spatially structured environmental filtering of collembolan traits in late successional salt marsh vegetation
Source: Oecologia. 2015 May 23;179(2):537–49. doi: 10.1007/s00442-015-3345-z (PMC4568007; doi:10.1007/s00442-015-3345-z)
Supplement: Supplementary file 2 — Supplementary material 2 (DOCX 42 kb) [file 442_2015_3345_MOESM2_ESM.docx]

**ELECTRONIC SUPPLEMENTARY MATERIAL (Online Resource 2)**

**Spatially structured environmental filtering of Collembola traits in late successional salt marsh vegetation**

Lina A. Widenfalk^1^, Jan Bengtsson^1^, Åsa Berggren^1^, Krista Zwiggelaar^2^, Evelien Spijkman^2^, Florrie Huyer-Brugman^2^ and Matty P. Berg^2, 3^

1. Department of Ecology, Swedish University of Agricultural Sciences, P.O. Box 7044, Uppsala SE-75007, Sweden
2. Department of Ecological Sciences, VU University, Amsterdam, De Boelelaan 1085, 1081 HV Amsterdam, The Netherland
3. Community and Conservation Ecology Group, Center for Ecological and Evolutionary Studies, University of Groningen, Nijenborgh 7, 9747 AG Groningen, The Netherlands

Corresponding author: Lina A. Widenfalk

e-mail: [lina.ahlback@slu.se](mailto:lina.ahlback@slu.se)

telephone: +46(0)18-67 20 21

Fax no: +46(0)18-672890

In addition to the PCNM analysis of variance partitioning and multiple regression presented in the paper, we performed an equivalent analysis using trend surface analysis as descriptor of the spatial configuration of the samples. This was done by centring by mean of X – Y coordinates (i.e. the relative position of all samples to each other) and adding a cubic trend surface regression, enabling more complex spatial trends than only linear gradients to be modelled (Borcard et al. 1992). Second-degree variables (X^2^, Y^2^, XY) represent hump-shaped responses and third-degree variables (X^2^Y, XY^2^, X^3^ and Y^3^) show if there are changes in the response variable that have another more complex structure (Legendre and Legendre 1998). For description of statistical procedures see Widenfalk (2014).

**Table ESM1** Variance in the composition of Collembolan species and community weighted mean trait values partitioned in environmental and spatial variables. The numbers given are the sum of all canonical eigenvalues (% of variation explained) obtained by the different sets from RDA analyses.

|  | Species | Traits |
| --- | --- | --- |
| Explained variation (E + S) | 28.1 | 45.4 |
| Pure spatial variation (S\|E) | 5.4 | 8.1 |
| Pure environmental variation (E\|S) | 20.0 | 32.2 |
| Shared variation (S ᴖ E) | 2.7 | 5.2 |
| Spatial variation (S) | 8.2 | 13.2 |
| Environmental variation (E) | 22.7 | 37.3 |
| Unexplained variation | 71.9 | 54.6 |

**Table ESM2** Environmental and spatial variables included in the RDAs describing A, species composition and B, CWM traits composition, in the order selected with forward selection based on Monte Carlo permutation test (999 perm). Before inclusion the moisture percentage was square root-transformed, while vegetation height and litter thickness were ln-transformed.

A, Species composition

| **Environmental variable** | **% variance explained** | **Inflation factor** |
| --- | --- | --- |
| 1. Topography | 14.5 | 1.5823 |
| 2. Moisture% | 3.3 | 1.5872 |
| 3. Vegetation height | 2.6 | 1.0392 |
| 4. Litter thickness | 1.3 | 1.2822 |

| **Spatial variable** | **% variance explained** | **Inflation factor** |
| --- | --- | --- |
| 1. Y^3^ | 1.9 | 8.6972 |
| 2. Y | 3.4 | 8.4166 |
| 3. X | 1.7 | 1.3945 |
| 4. X^2^ | 1.2 | 1.2291 |

B, Trait CWM composition

| **Environmental variable** | **%variance explained** | **Inflation factor** |
| --- | --- | --- |
| 1. Topography | 20.8 | 1.5435 |
| 2. Moisture% | 6.2 | 1.5802 |
| 3. Vegetation height | 5.7 | 1.0232 |
| 4. Litter thickness | 4.6 | 1.2979 |

| **Spatial variable** | **% variance explained** | **Inflation factor** |
| --- | --- | --- |
| 1. Y | 3.0 | 8.4215 |
| 2. Y^3^ | 5.7 | 8.6992 |
| 3. X^2^ | 1.8 | 1.3896 |
| 4. X^3^ | 2.7 | 1.4993 |

**Variance explained:** indicates how much additional variation is explained when the given variable is added to the model (in the order shown in the table). This is done for the environmental and spatial variables separately. **Inflation factor**: indicates how much the variables are correlated to each other. These values are from the final model with four environmental and four spatial variables included. An inflation factor value >20 indicates that the variable is almost perfectly correlated with the other variables and therefor has no unique contribution to the regression equation (ter Braak and Smilauer 2002).

**Fig. ESM1** The proportion of variation in single trait community weighted mean (CWM), explained by spatial and environmental variables, respectively. Separate regressions for each CWM. Explanatory variables included, R^2^-values and significance of each model are shown in Table ESM2.

**Table ESM2** Summary of multiple linear regressions of all single trait CWM, and environmental and spatial variables (as described by a polynomial combination of X- and Y-coordinates). *** P < 0.001

| Trait | Significant variables including direction of correlation | | Adj R^2^ |
| --- | --- | --- | --- |
|  | **Environmental** | **Spatial** |  |
| Body length^***^ | Topography (+)  Soil moisture (-)  Vegetation height (-)  Litter thickness (-)  Litter mass (-) | Y^3^ (+)  XY^2^ (+)  Y (-) | 0.470 |
| Antenna/body ratio^***^ | Vegetation height (-)  Topography (+)  Soil moisture (-)  Litter mass (-)  Litter thickness (-) | Y^3^ (+)  (XY^2^) (+)  Y (-) | 0.431 |
| Life form^***^ | Topography (+)  Soil moisture (-)  Litter thickness (-)  Vegetation height (-)  Litter mass (-) | Y^3^ (+)  (XY) (+)  Y (-)  XY^2^ (+) | 0.557 |
| Moisture preference^***^ | Vegetation height (-)  Topography (-)  (Litter mass) (-) | Y (+)  X (+)  (X^2^Y) (-) | 0.321 |
| Habitat width^***^ | Topography (+)  Litter thickness (+)  Soil moisture (-) | (Y^3^) (+)  X (+) | 0.327 |

Shown are all variables included in the final multiple regression model, after stepwise selection (based on *P*-values). Variables that did not contribute significantly (P > 0.5) to the model are within brackets. After each variable the direction of the estimate (positive or negative) in the final model is given. Variables are shown in order of amount of variance explained when evaluating each variable separately in linear regressions.

**References:**

Borcard D, Legendre P, Drapeau P (1992) Partialling out the spatial components of ecological variation. Ecology 73:1045-1055

Legendre P, Legendre L (1998) Spatial analyses. Numerical ecology - developments in environmental modelling 20. Elsevier, Amsterdam. pp 707-786

ter Braak CJF, Smilauer P (2002) CANOCO Reference manual and CanoDraw for Windows User's guide: Software for Canonical Community Ordination (version 4.5). Microcomouter Power, Ithaca, NY, USA

Widenfalk LA (2014) Traits or species – space or environment: how to understand the spatial structure of springtail community composition. PhLic dessertation. Department of Ecology, Swedish University of Agricultural Sciences, Uppsala
